# Supplementary figures and images for: Low-dose hexavalent chromium induces mitophagy in rat liver via the AMPK-related PINK1/Parkin signaling pathway
Source: PeerJ. 2024 Jul 30;12:e17837. doi: 10.7717/peerj.17837 (PMC11296300; doi:10.7717/peerj.17837)

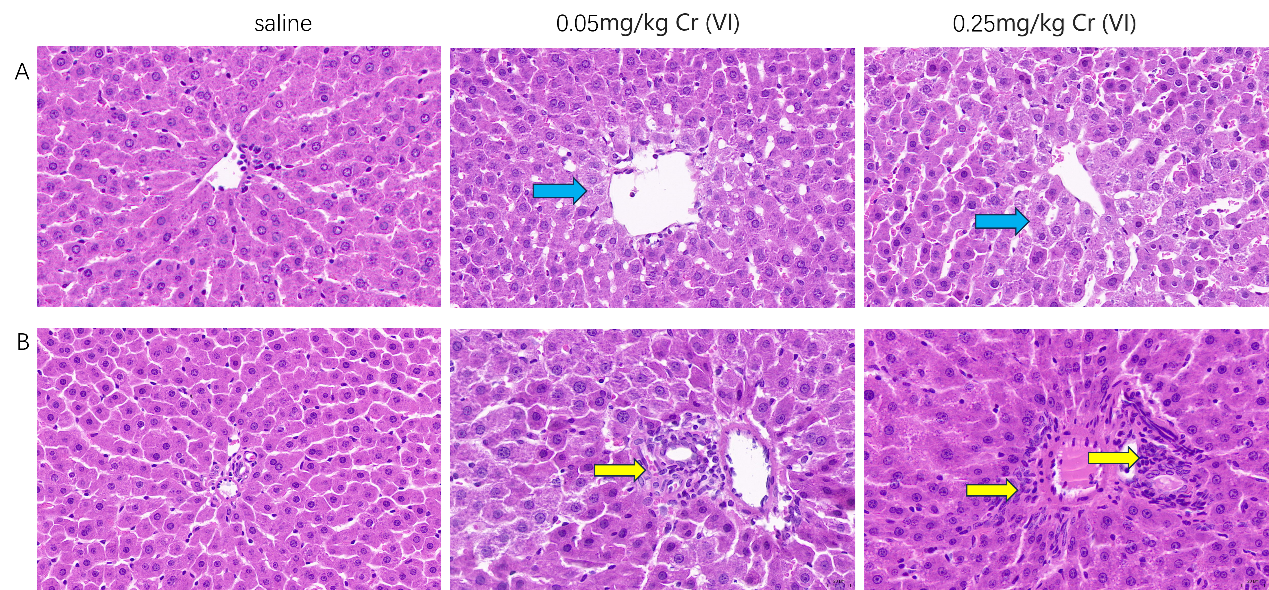

Supplement: Supplemental Information 3 — (A). pathological changes of liver tissue (Central vein). (×400 magnification) (B). pathological changes of liver tissue (portal area). (×400 magnification) Blue arrows indicate edematous degeneration. Yellow arrows indicate necrosis and inflammatory cell infiltration [file peerj-12-17837-s003.png]
